# Whole-genome sequencing of *Ganoderma boninense*, the causal agent of basal stem rot disease in oil palm, via combined short- and long-read sequencing

**DOI:** 10.1038/s41598-024-60713-3

**Published:** 2024-05-08

**Authors:** Condro Utomo, Zulfikar Achmad Tanjung, Redi Aditama, Antonius Dony Madu Pratomo, Rika Fithri Nurani Buana, Hadi Septian Guna Putra, Reno Tryono, Tony Liwang

**Affiliations:** 1 Department of Biotechnology, PT SMART Tbk, Bogor, 16810 Indonesia; 2 Section of Bioinformatics, PT SMART Tbk, Bogor, 16810 Indonesia; 3 Section of Clonal Technology, PT SMART Tbk, Bogor, 16810 Indonesia; 4 Section of Microbiome Technology, PT SMART Tbk, Bogor, 16810 Indonesia; 5 Section of Data Processing, PT SMART Tbk, Bogor, 16810 Indonesia; 6 Section of Genetic Engineering, PT SMART Tbk, Bogor, 16810 Indonesia; 7 Division of Plant Production and Biotechnology, PT SMART Tbk, Bogor, 16810 Indonesia

**Keywords:** Chromosomes, Genomics, Sequencing, Agricultural genetics

## Abstract

The hemibiotrophic Basidiomycete pathogen *Ganoderma boninense* (*Gb*) is the dominant causal agent of oil palm basal stem rot disease. Here, we report a complete chromosomal genome map of *Gb* using a combination of short-read Illumina and long-read Pacific Biosciences (PacBio) sequencing platforms combined with chromatin conformation capture data from the Chicago and Hi-C platforms. The genome was 55.87 Mb in length and assembled to a high contiguity (N50: 304.34 kb) of 12 chromosomes built from 112 scaffolds, with a total of only 4.34 Mb (~ 7.77%) remaining unplaced. The final assemblies were evaluated for completeness of the genome by using Benchmarking Universal Single Copy Orthologs (BUSCO) v4.1.4, and based on 4464 total BUSCO polyporales group searches, the assemblies yielded 4264 (95.52%) of the conserved orthologs as complete and only a few fragmented BUSCO of 42 (0.94%) as well as a missing BUSCO of 158 (3.53%). Genome annotation predicted a total of 21,074 coding genes, with a GC content ratio of 59.2%. The genome features were analyzed with different databases, which revealed 2471 Gene Ontology/GO (11.72%), 5418 KEGG (Kyoto Encyclopedia of Genes and Genomes) Orthologous/KO (25.71%), 13,913 Cluster of Orthologous Groups of proteins/COG (66.02%), 60 ABC transporter (0.28%), 1049 Carbohydrate-Active Enzymes/CAZy (4.98%), 4005 pathogen–host interactions/PHI (19%), and 515 fungal transcription factor/FTFD (2.44%) genes. The results obtained in this study provide deep insight for further studies in the future.

## Introduction

The oil palm of African origin, *Elaeis guineensis*, is the most productive oil-bearing crop in the world. This species was introduced by the Dutch into Indonesia in 1848 as an ornamental palm at the Bogor Botanical Garden^1^. It has become a fundamental oil palm ancestor of the current massive cultivation of this plant species in Southeast Asia, particularly Indonesia and Malaysia. This oil crop produces an average of 3.5 tons of oil per hectare, although it has a full yield potential of up to 18 tons/ha/year from the third year onward^2^. Moreover, with its current status, palm oil contributes almost one-third (75.17 million tons) of the world’s total vegetable oils^3^. The oil benefits the food, oleochemical, and renewable energy source sectors^4^. However, the industry is facing serious yield limitations due to basal stem rot (BSR) disease.

**Figure 1:**
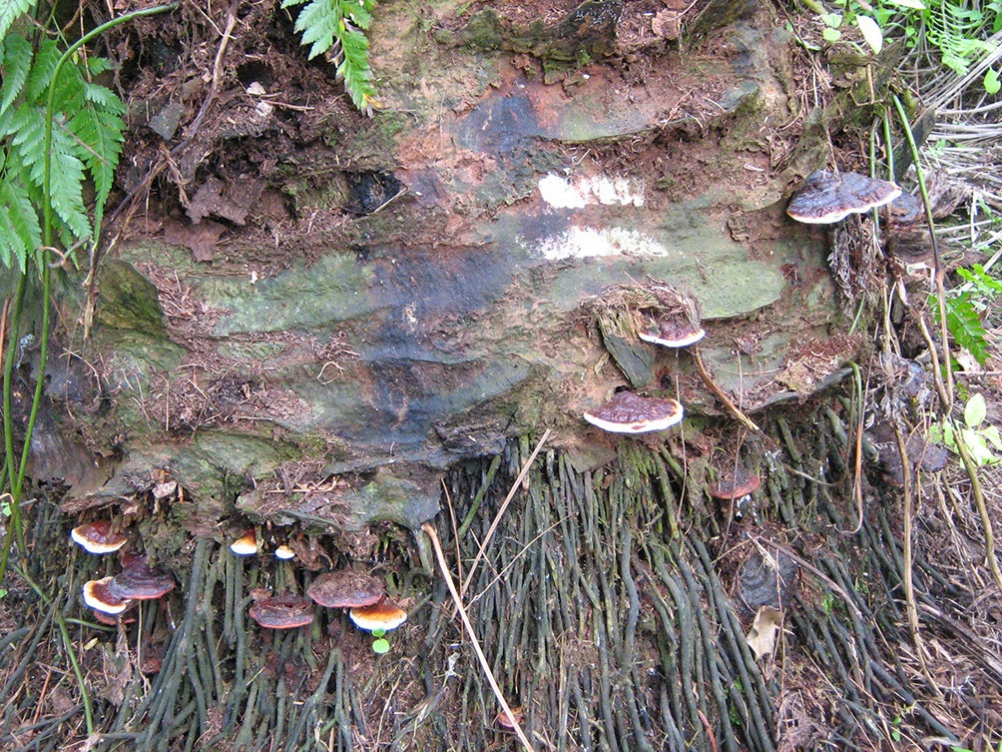
BSR incidence in the field showing fruiting bodies of *Gb* emerging from the basal stem of an oil palm tree.

BSR is associated with 15 *Ganoderma* spp. (Basidiomycota: Polyporales) strains worldwide^5^. However, pathogenic interactions with oil palm have been reported for only three species, i.e., *G. boninense* (*Gb*), *G. zonatum*, and *G. miniatocinctum*^6,7^. Although *Gb* is a dominant pathogen for BSR, the latter two species have rarely been reported as causal agents of BSR cases in the field^8,9^ (Fig. 1). Nevertheless, BSR remains a major economically important problem in the oil palm industry in Indonesia, Malaysia and Papua New Guinea^10–12^. Despite deep interest in fungi, there is little genetic and genomic information available to provide insights into host-interactions^13–15^. On the other hand, a complete genome sequence of another *Ganoderma* fungus, *G. lucidum* (*Gl*), revealed remarkable gene cooperation for secondary metabolite biosynthesis as well as the richest sets of wood degradation enzymes among all sequenced basidiomycetes^16^. Interestingly, *Gl* has been famous for its edible pharmaceutical herb value for thousands of years^17,18^, rather than being a hemibiotroph phytopathogen, as shown by *Gb*^19,20^. Therefore, in this study, a genome comparison analysis might provide insight into the evolutionary histories, scenes and trajectories of both fungi, which impart speciation and lifestyle.

Recent advances in sequencing technologies have made the full genome assembly of interesting species relatively affordable. At least eight commercial sequencing platforms are available, despite each having their own merits, whereas one platform could be better fit for a study than another depending on the purpose^21^. In general, these platforms are based mainly on short- and long-read sequencing approaches in which the divergences are related to the throughput, cost, error rate, and read structure^22^. The combination of short- and long-read sequencing approaches, such as the Illumina HiSeq 4000 and Pacific Bioscience platforms, was used to generate a high-quality whole-genome sequence and assembly of *Auricularia heimuer* (Basidiomycota: Auriculariales) and *Arthrinium phaeospermum* (Ascomycota: Sordariomycetidae)^23,24^. Moreover, the birth of the 3C (chromosome conformation capture) technique in 2002 involving formaldehyde crosslinking of nuclei provides pairwise linking information between reads that originate from genomic regions that are physically adjacent in a nucleus^25^. The combination of Illumina and PacBio sequencing coupled with the two most common 3C derivative protocols, Chicago and Hi-C, allows the collection of fragments/bins/genomic loci that are profiled as read-pair interactions on an “all-by-all” basis in the entire genome^26–28^. In addition, the HiRise™ software pipeline builds an accurate and contiguous genome assembly, identifies poor-quality joins and produces accurate, long-range sequence scaffolds^29^. Proximity-based ligation and massive sequencing of reads form a three-dimensional architecture of chromosomes (Hi-C) with a high probability of intrachromosomal contacts, even when separated by > 200 Mb, to generate a reasonably accurate chromosome-scale de novo assembly^30,31^. Here, we report the de novo genome assembly of *Gb* using Illumina short reads and PacBio long reads coupled with the Chicago + HiRise assembler to construct scaffolds, and the resulting scaffolds were subsequently aligned to the Hi-C + HiRise platform to generate chromosome levels.

## Results

### Global genome assembly and characteristics

The genome sequence of the *Gb* strain G3 was used in this study^32,33^. A fine draft genome sequence was constructed by the hybrid assembly of short reads on the Illumina HiSeq 4000 platform combined with long reads on the PacBio RS II platform to generate a draft genome of this fungus. The libraries from both platforms were assembled using WTDBG2^34^, followed by short- and long-read iterative correction using each two-round Racon and Pilon as the polishers constitutively^35,36^. The hybrid sequences from the two platforms were assembled de novo to construct a 55.82 Mb total draft genome sequence with 826x sequencing coverage, containing 592 genomic scaffolds with an N50 length of 0.357 Mb.

In the downstream scaffolding, the contigs were scaffolded in a series of HiRise analyses, initially using the Chicago data followed by inclusion of the Hi-C reads for chromosome-scale assembly of *Gb*. Using the Chicago approach, the library produced 165 million reads of 2 × 150 bp or provided 1887.11× physical coverage of the genome. The Hi-C library produced 194 million read pairs of 2 × 150 bp or provided 41,650.61× physical coverage of the genome. Using Illumina and PacBio reads as input assembly data, the Chicago HiRise pipeline for assembly resulted in a 55.86 Mb total assembled sequence with 264 scaffolds. This result was used as an input assembly for the HiRise assembler and resulted in a final 112 scaffolds with a total genome assembly length of 55.87 Mb (Table 1). The scaffold joined by the Hi-C mate pairs introduced new “N” gaps in the assembly, thereby increasing the number of gaps in the assembly to 7201, and each gap consisted of 100 “N”. The final genome assembly was characterized by 21,074 coding genes, with a GC content ratio of 59.2%. The average gene length was 1265 bp, while the average intergenic size was 1250 bp. The average number of exons per gene was 7, with average exon and intron sizes of 178 and 97 bp, respectively (Table 2).

**Table 1:** Assembly statistics based on next-generation sequencing technologies and software assemblers.

Assembly	Software	Assembly levels	Longest scaffold (Kb)	Number of scaffolds	N50 (Mb)	L90	Assemble genome (Mb)
Illumina + PacBio	WTDBG2 + Racon + Pilon	Contigs	1,682	592	0.357	257	55.82
PacBio* + Chicago	HiRise	Scaffolds	3,150	264	0.993	75	55.86
PacBio* + Chicago + Hi-C	HiRise	Chromosomes	6,651	112	4.126	12	55.87

*PacBio: PacBio platform polished by Illumina.

**Table 2:** Genome characteristics of *Gb.*

Characteristics	Number
Chromosome	12
L50 (scaffolds)	6
N90 scaffold (Mb)	3.032
GC contents (%)	56.2
Protein-coding genes	21,074
GC content of protein-coding genes (%)	59.2
Average gene length (bp)	1265
Average exons per gene	7
Average exon size (bp)	178
Average intron size (bp)	97
Average size of intergenic regions (bp)	1250
Gaps	7201
Nongap bases (bp)	55,821,405
Gap bases (bp)	720,100

**Figure 2:**
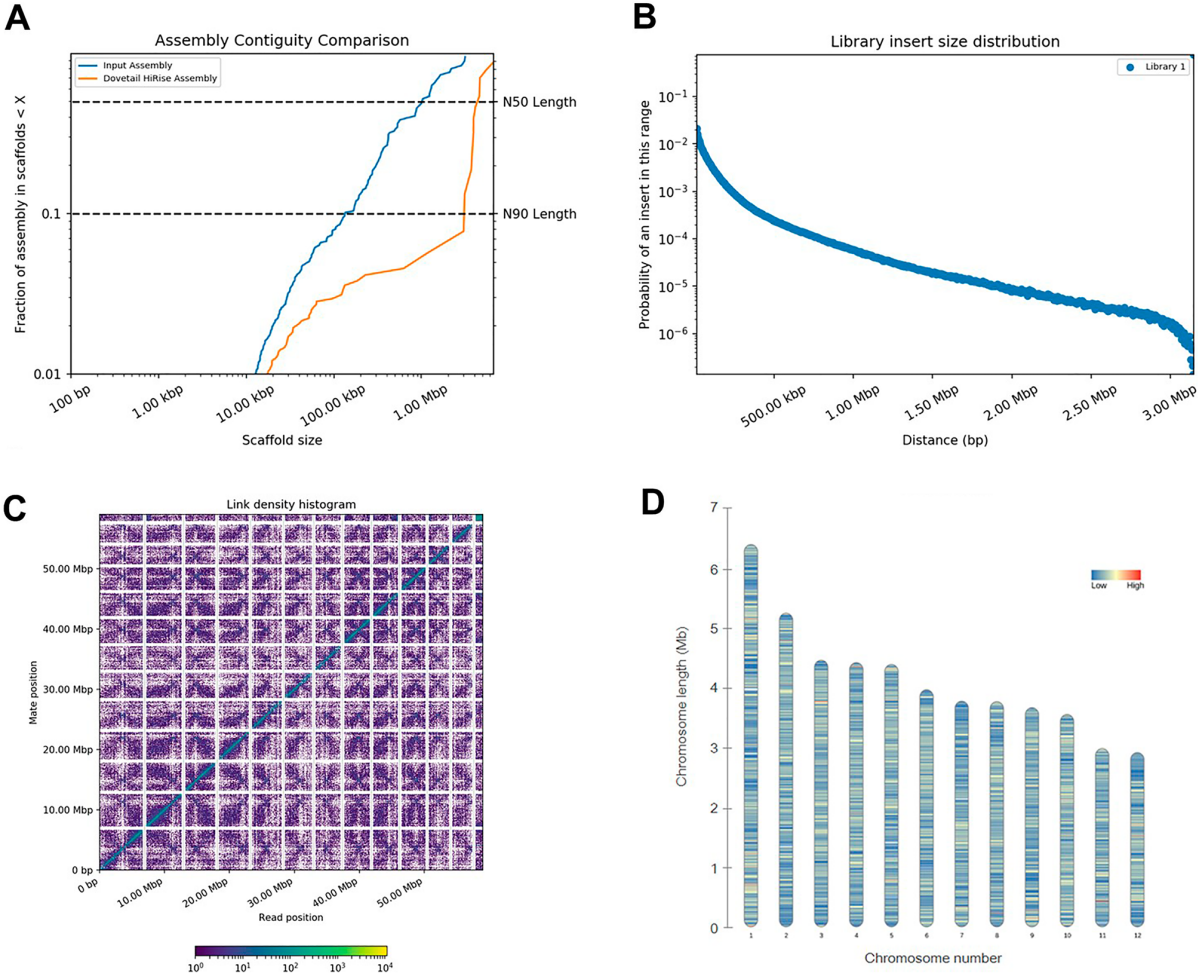
Chromosome assembly of *Gb* via dovetail genomics. (**A**) Comparison of the contiguity of the input assembly and the final HiRise scaffolds. Each curve shows the fraction of the total length of the assembly present in scaffolds of a given length or shorter. The fraction of the assembly is indicated on the Y-axis, and the scaffold length in base pairs is given on the X-axis. The two dashed lines mark the N50 and N90 lengths of each assembly. (**B**) The distribution of insert sizes. The distance between the forward and reverse reads is given on the X-axis in base pairs, and the probability of observing a read pair with a given insert size is shown on the Y-axis. (**C**) Hi-C linkage density histogram. In this figure, the x- and y-axes show the mapping positions of the first and second reads in the read pair, respectively, which are grouped into bins. The color of each square indicates the number of read pairs within that bin. Dots (sequences) within boxes in the last column are probably 4.34 Mb of unassembled scaffold DNA sequence with only ~ 7.77% bases. White vertical and black horizontal lines have been added to show the borders between the scaffolds. (**D**) Chromosome structures illustrating the gene density on each chromosome.

The contiguity comparison graph showed a significant improvement in the final assembly (Fig. 2A), and the library insert size distribution graph exhibited an accurate distribution of reads to facilitate the repeat bridging and resolution capabilities of the state-of-the-art assembly (Fig. 2B). In addition, the HiRise pipeline generated a Hi-C linkage density histogram (Fig. 2C). The alignment produces a diagonal of lines from the lower left to upper right in the plot that represent each of the 12 *Gb* chromosome-scale scaffolds ranging from 3.03 to 6.65 Mb (Fig. 2D, Table S1).

**Figure 3:**
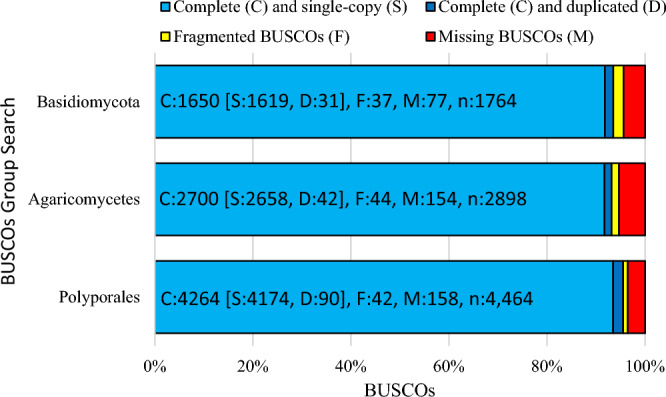
Three BUSCO scores of the Polyporales, Agaricomycetes, and Basidiomycota group searches illustrating the assembly and annotation completeness of the *Gb* genome.

The final assemblies were evaluated for genome completeness by using BUSCO^37^ version 4.1.4 based on 4464 total BUSCO polyporales group searches. The assemblies yielded 4264 (95.52%) of the conserved orthologs as complete common eukaryote gene sequence coverage, with only a few missing (158, or 3.53%), as well as a duplicate score of 90 (2.01%) and a fragmented BUSCO of 42 (0.94%). In addition, BUSCO analysis of Agaricomycetes and Basidiomycota group searches was also conducted, and the results were similar. The completeness levels were 93.16% and 93.53% for the two groups, respectively (Fig. 3, Table S2). This result indicates that our genome assembly is of relatively high quality and contiguous.

Genome assembly could be an issue in diploid or polyploid samples due to possible discrepancies within reads or contigs that arbitrarily alternate between parental alleles for certain genomic regions. Indeed, our assembly revealed the extent of natural heterozygosity in the diploid *Gb* mycelial samples used for sequencing. De novo assemblies were used to merge homologous and heterozygous loci into single “consensus” sequences. Variant analysis in this study detected 527,941 high-confidence polymorphisms for the entire genome by aligning sequence reads to the total assembled *Gb* genome and calling variants, including insertions-deletions (indels) and nucleotide polymorphisms (SNPs). We found, on average, 9 variants in every 1 kb of the *Gb* genome (Table S3). However, in this study, the BUSCO analysis results demonstrated the intact de novo assembly of *Gb* from a diploid material genetic source.

### Gene prediction and functional annotation

Among the 21,074 annotated genes in the *Gb* genome, we characterized their functions using different databases, including the Gene Ontology (GO), Kyoto Encyclopedia of Genes and Genomes (KEGG) Orthologous (KO), Cluster of Orthologous Groups of Protein (COG), ATP-Binding Cassette (ABC) Transporter, Carbohydrate-Active Enzyme (CAZy), Pathogen-Host Interaction (PHI), and Fungal Transcription Factor (FTFD) databases. GO analysis has become the most complete, comprehensive, and wide-ranging method for predicting gene function in various eukaryotic species. In this study, the GO database annotated 2471 coding genes in *Gb* categorized into three major classes, i.e., biological process, cellular component, and molecular function. Biological process was the most enriched function, with 25,648 annotations, followed by cellular component and molecular function, with 12,494 and 9976 annotations, respectively (Table S4). Most of the biological processes were annotated as proteolysis (GO:0006508), with 108 (0.42%) annotations, while most of the cellular component and molecular function terms were related to protein binding (GO:0005515) and cytosol (GO:0005829), with 591 (5.66%) and 841 (4.92%) annotations, respectively.

**Figure 4:**
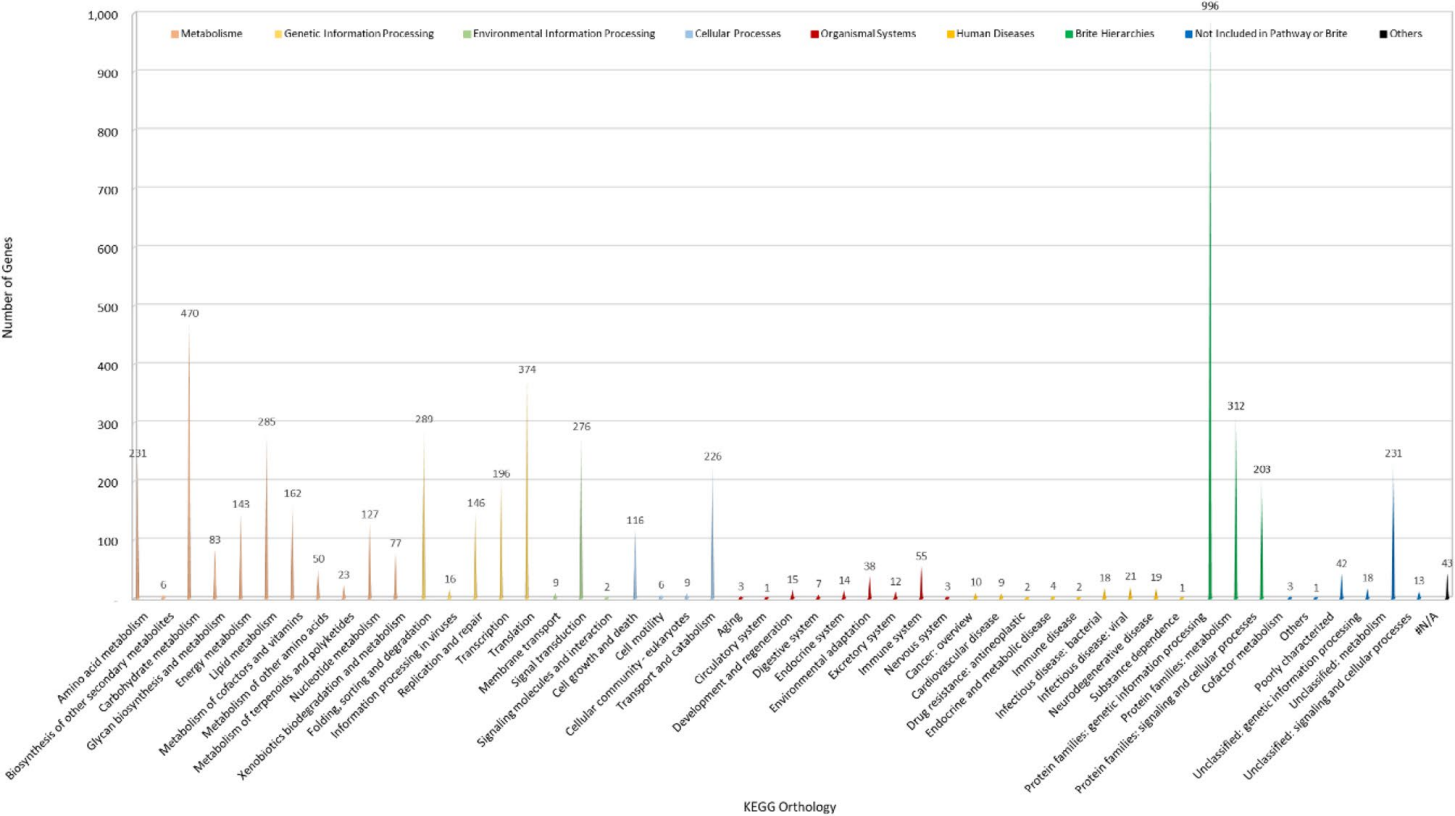
The KO functional annotation of the *Gb* genome is shown. The major classes and the number of genes from the subclass partitions of interest are represented. The X-axis represents the scale for the number of genes. KEGG functional annotation was divided into nine major classes: cellular process, environmental information processing, genetic information processing, human diseases, metabolism or organismal system, and 40 subclasses. Each class is represented by a distinct color.

**Figure 5:**
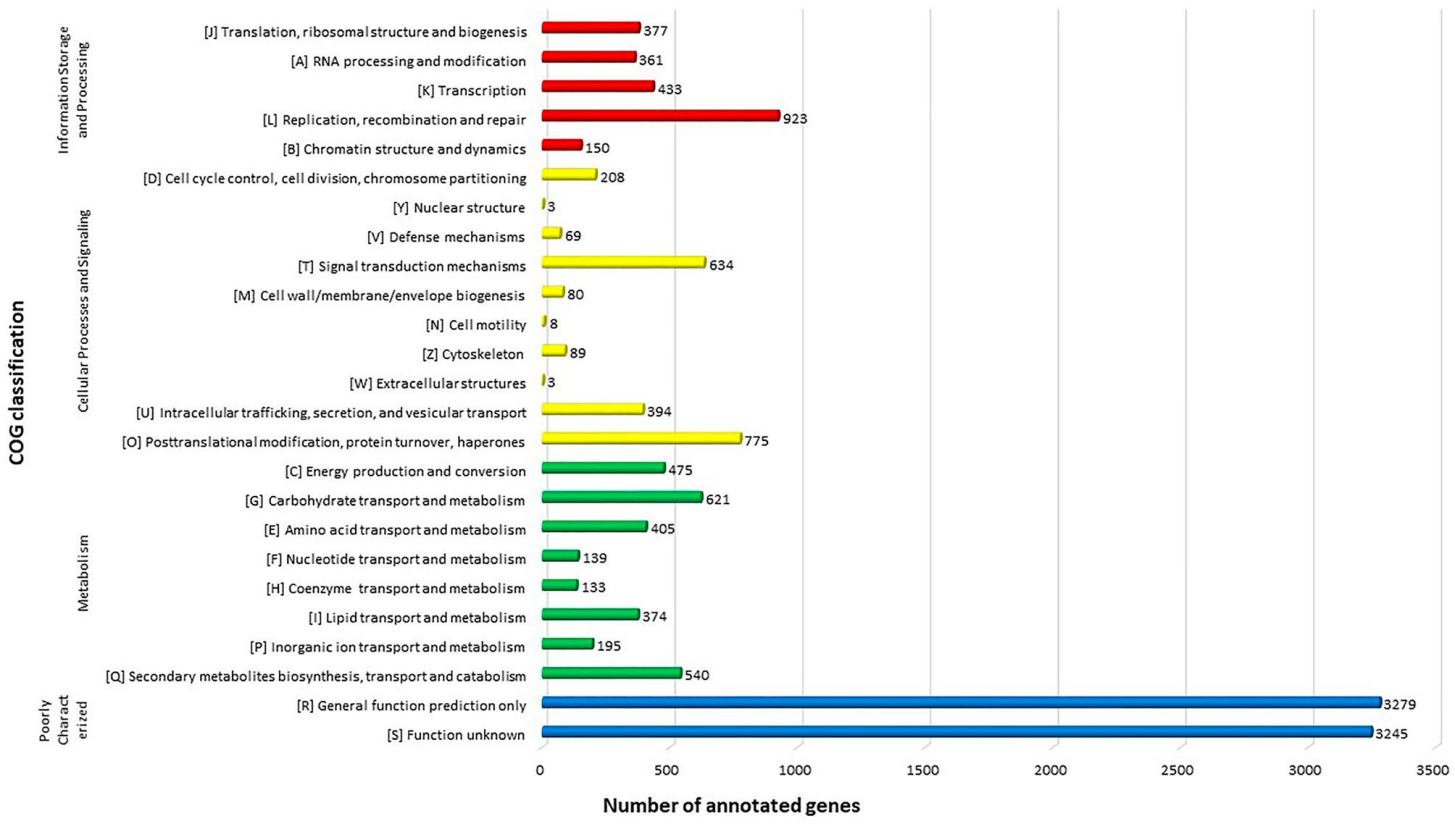
COG functional classification of proteins in the *Gb* genome. The X-axis represents the scale for the number of genes. COG functional annotation is divided into four classes: information storage and processing, cellular process and signaling, metabolism, and poorly characterized. Each class is represented by a distinct color.

Subsequently, 25.71% (5418) of the *Gb* coding sequences were annotated via the KO database. The biological pathways are divided into nine KO categories, each of which is subdivided, and each category is labeled with the relevant information. Among these predicted genes, those involved in the metabolism function category accounted for the majority (30.58%, 1657) of the annotated genes. In contrast, those categorized as “others” were the least annotated at 0.79% (43) (Fig. 4, Table S5). We also enriched all the coding sequences of the assembled *Gb* genome with the COG database. The functional analyses revealed that 66.02% (13,913 genes) of the coding sequences were annotated. However, almost half (6524) of the annotations were categorized as poorly categorized. This might be because the database was built from predominantly unicellular microorganisms of 1187 bacterial and 122 archaeal genomes and only seven sequenced eukaryotic genomes, including those of *Homo sapiens*, *Drosophila melanogaster*, *Caenorhabditis elegans*, *Arabidopsis thaliana*, *Saccharomyces cerevisiae*, *Schizosaccharomyces pombe*, and *Encephalitozoon cuniculi*^38,39^. Moreover, those with known functions are relatively common in all living organisms, and their abundance is equally distributed among the three COG categories, such as information storage and processing, cellular process and signaling, and metabolism (Fig. 5 top, Table S6).

**Figure 6:**
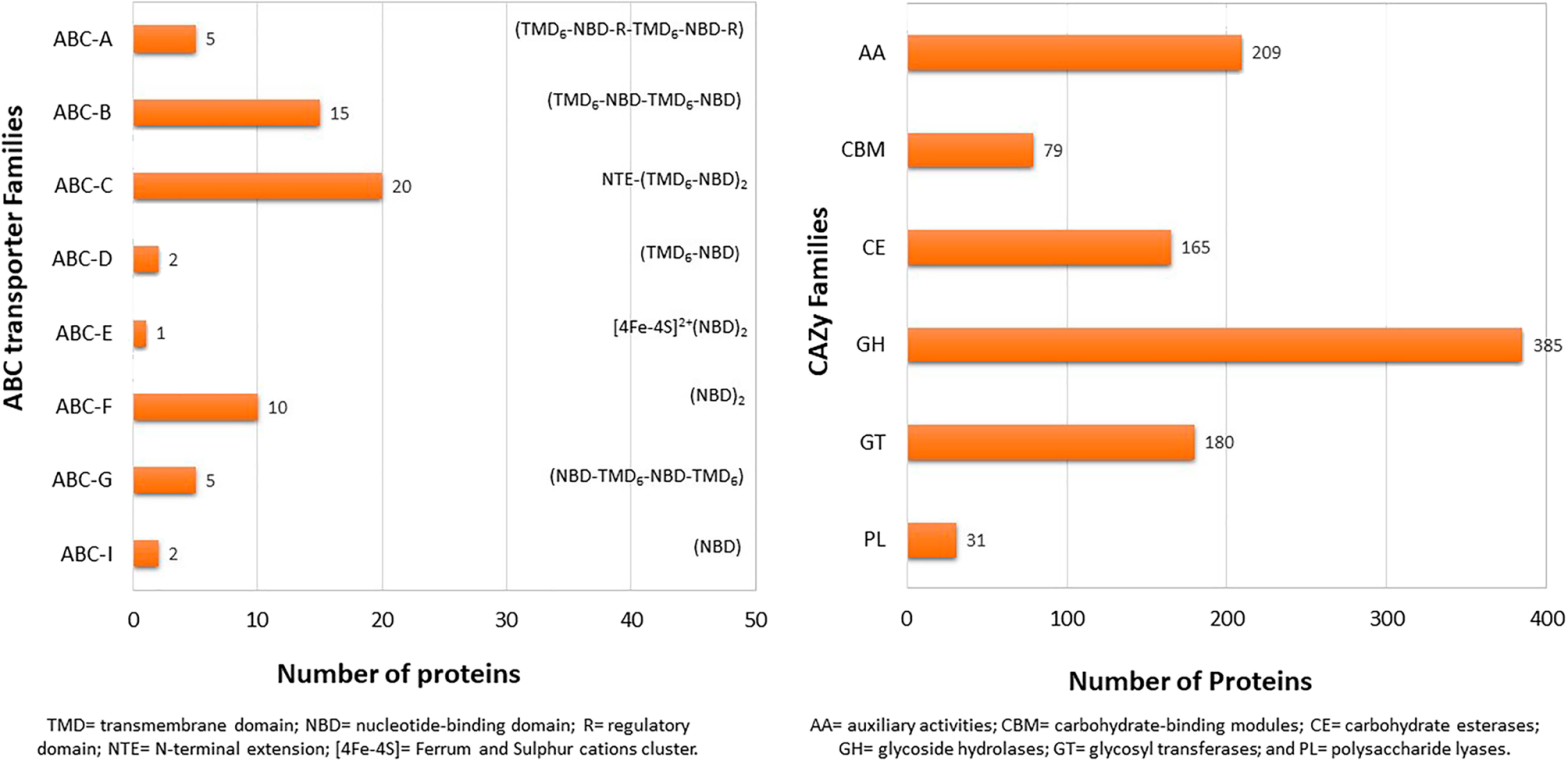
The distribution of ABC transporters and CAZy families in the *Gb* genome. Left. The ABC family parentheses indicate structural component domains in each ABC family. B. The abundance of CAZys for each family in the genome.

In other analyses, we also identified the presence of ABC transporter proteins in the *Gb* genome that actively transport a wide substrate repertoire and range of functions. A total of 60 predicted genes were found, which were distributed in eight families, whereas the full-length multidrug resistance protein (MRP/ABC-C) family contributed the most genes (Fig. 6-left, Table S7). Additionally, CAZy, a carbohydrate-degrading enzyme, is a dominant virulence factor in white-rot Basidiomycetes. Here, our CAZy prediction revealed 1049 encoding genes in the *Gb* genome, accounting for 4.98% of all annotated genes. Among the classes, glycoside hydrolases (GHs) were the most common, with 385 genes (37%), followed by genes involved in auxiliary activities (AAs), glycosyl transferases (GTs), carbohydrate esterases (CEs), carbohydrate-binding modules (CBMs), and polysaccharide lyases (PLs) (Fig. 6-right, Table S8).

**Figure 7:**
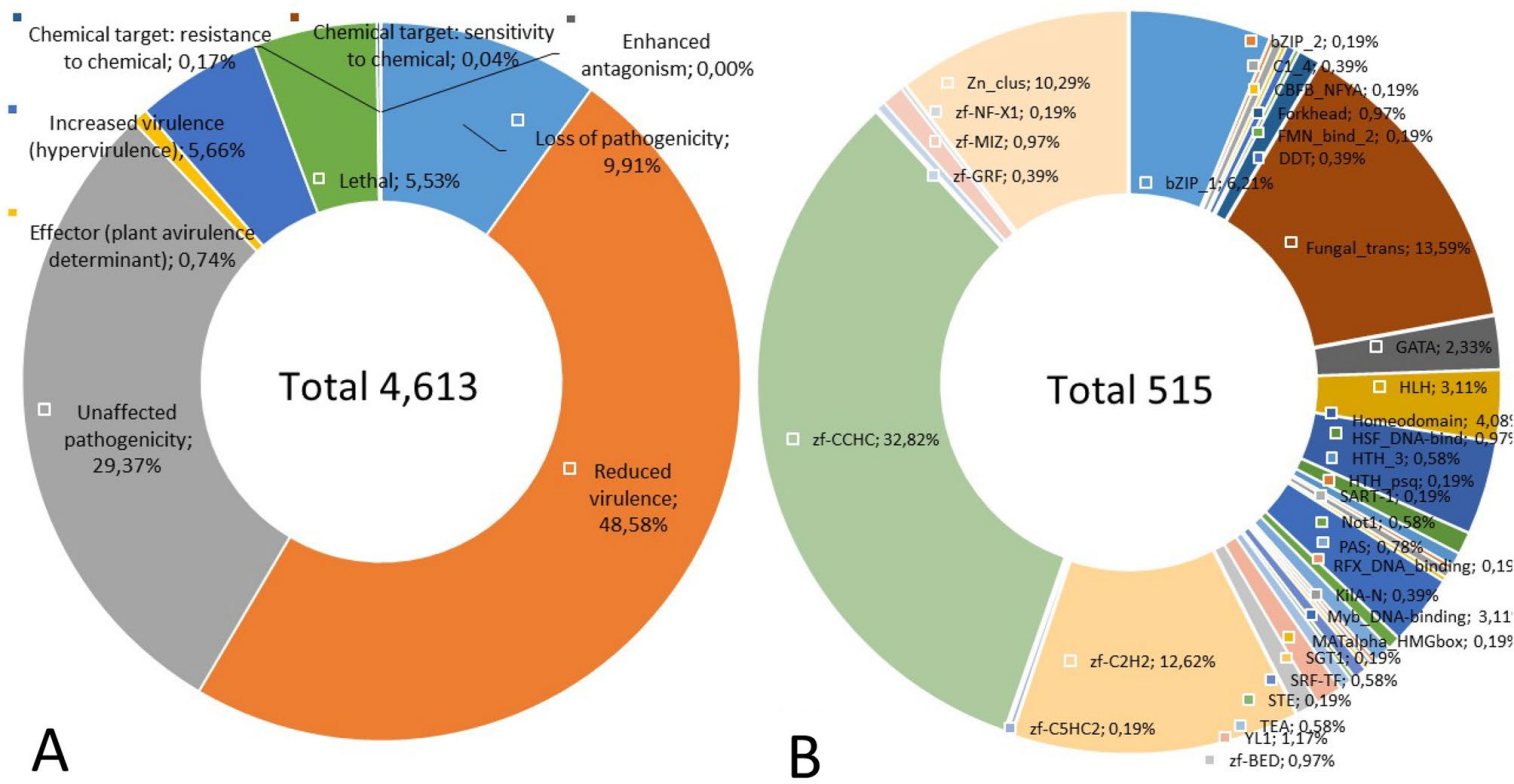
The abundance of predicted PHI phenotypic-category genes and distribution of transcription factor families in *Gb*. The percentage of each PHI category (**A**) and TF family (**B**) relative to the total number of predicted genes.

Moreover, we found a total of 4005 predicted *Gb* genes associated with pathogen-host interactions (PHIs). Among those, 4613 were annotated as the highest proportion, as high as 48.58% (2241), which was related to reduced virulence, followed by unaffected pathogenicity (29.37%), loss of pathogenicity (9.91%), increased virulence (5.66%), lethality (5.53%), and others below 1% (Fig. 7A, Table S9). Transcription factors (TFs) are important regulators of gene expression for modulating diverse biological processes, and some have been reported to act as virulence factors for fungal phytopathogens^40^. In this study, we reported that 515 genes encoding TFs were identified in the *Gb*, accounting for 2.84% of the total number of predicted genes. The largest class was the zinc finger CCHC (pfam domain: PF00098.26), with 169 members or 32.82% of the total TFs. This is followed by fungal trans (pfam domain: PF04082.21) and zinc finger C2H2 (pfam domain: PF00096.29), with 70 and 65 members, or 13.59% and 12.62%, respectively. The other classes were distributed at 10% and below (Fig. 7B, Table S10).

## Discussion

*Ganoderma* Karst. (Ganodermataceae, Polyporales) is a cosmopolitan genus of white-rot fungi with more than 200 members^41,42^. Above, we reported high-quality chromosomal genome sequence assemblies of *Gb,* a white-rot fungal member that plays an ecological niche and is an economically important plant pathogen^1,10,43^, whereas BUSCO analysis supported its completeness and accuracy in ensuring the reliability of the gene structures^37^. The genome capacity and number of *Gb* genes are slightly greater than the average of all available Basidiomycota fungal whole-genome information, i.e., 55.87 out of 46.48 Mb and 21,074 out of 15,431 genes, respectively^44^. The available genome size of Basidiomycota fungi ranges from 9.82 (*Wallemia sebi*) to 130.65 Mb (*Dendrothele bispora*), even though the latest genome sequence project for some edible mushrooms has expanded in size to 202.2 Mb^45^. The revolution of sequencing and genome assembly technology paves the way for genomics studies of species of interest to reveal their indispensable genomic structure. Chromosome conformation capture techniques, such as the Hi-C and Dovetail Genomics Chicago libraries, in combination with long-read library sequencing platforms help span and resolve repetitive regions, thus greatly improving genome assembly^46^. In particular, Hi-C sequencing is considered an economical method for generating chromosome-scale scaffolds and for genome reconstruction^47^. The Hi-C assay is based on in vivo proximity ligation, along with the following principles: (1) Hi-C interactions tend to occur within the same chromosome because chromosomes occupy distinct territories and spatial preferences in the nucleus. (2) Hi-C separates nuclei after proximity ligation because genomic DNA is fixed within an intact nucleus, and cross-linking between nuclei is rare. (3) The Hi‒C interaction frequency exhibits genomic distance-dependent decay because according to the principles of polymer physics, cis-loci in linear sequences interact more frequently than trans-loci^48^. It is thus reliable to use Hi-C data to determine that contigs with frequent interactions are likely to belong to the same chromosome^31,49,50^.

In principle, we generated sufficient PacBio sequencing coverage to improve the scaffold N50 length, polished the assembly with Illumina short reads, and further used data from Dovetail Genomics Chicago and Hi‐C libraries to extend the scaffolds to the chromosomal level. The availability of long-read PacBio sequencing technology paves the way for achieving longer contigs with better quality assemblies, especially for the sequencing of large repeat regions that are not properly assembled or missed using short-read-based methods. Long-read sequencing coupled with Hi-C library technology is currently one of the best approaches for generating high-quality chromosome levels without knowledge of chromosome numbers. Dovetail™ Hi-C data can provide links across a variety of length scales, spanning even whole chromosomes, and this technique has been used to improve draft genome assemblies and create chromosome-length scaffolds for large genomes. The Dovetail Chicago and Hi‐C libraries increased the longest scaffold by more than threefold, from 1.6 to 6.6 Mbp or one-eighth of the total *Gb* genome, and the scaffold N50 increased by more than 11‐fold, from 0.36 to 4.13 Mbp, which means that the contiguity significantly improved. Another metric for assessing the quality of assemblies is the completeness of the so-called BUSCO. A “good” genome assembly criterion is expected to contain approximately 90% of the BUSCO gene set^51^. In this assessment, the BUSCO completeness scores of 98.56%, 93.53%, and 93.16% for Polyporales, Basidiomycota and Agaricomycetes, respectively, indicate that the current assembly is of high quality and highly consistent. In the future, other techniques, including optical mapping from BioNano^52^ combined with PacBio, may further improve the assembly quality, even though the joint accuracy of BioNano is reported to be 15% greater in Chicago^53^. In this approach, the methodology described here makes it feasible to obtain a near-finished or completely assembled *Gb* genome, which is reflected in the ability to obtain 12 scaffolds that correspond to 12 chromosomes in *Gb.*

**Figure 8:**
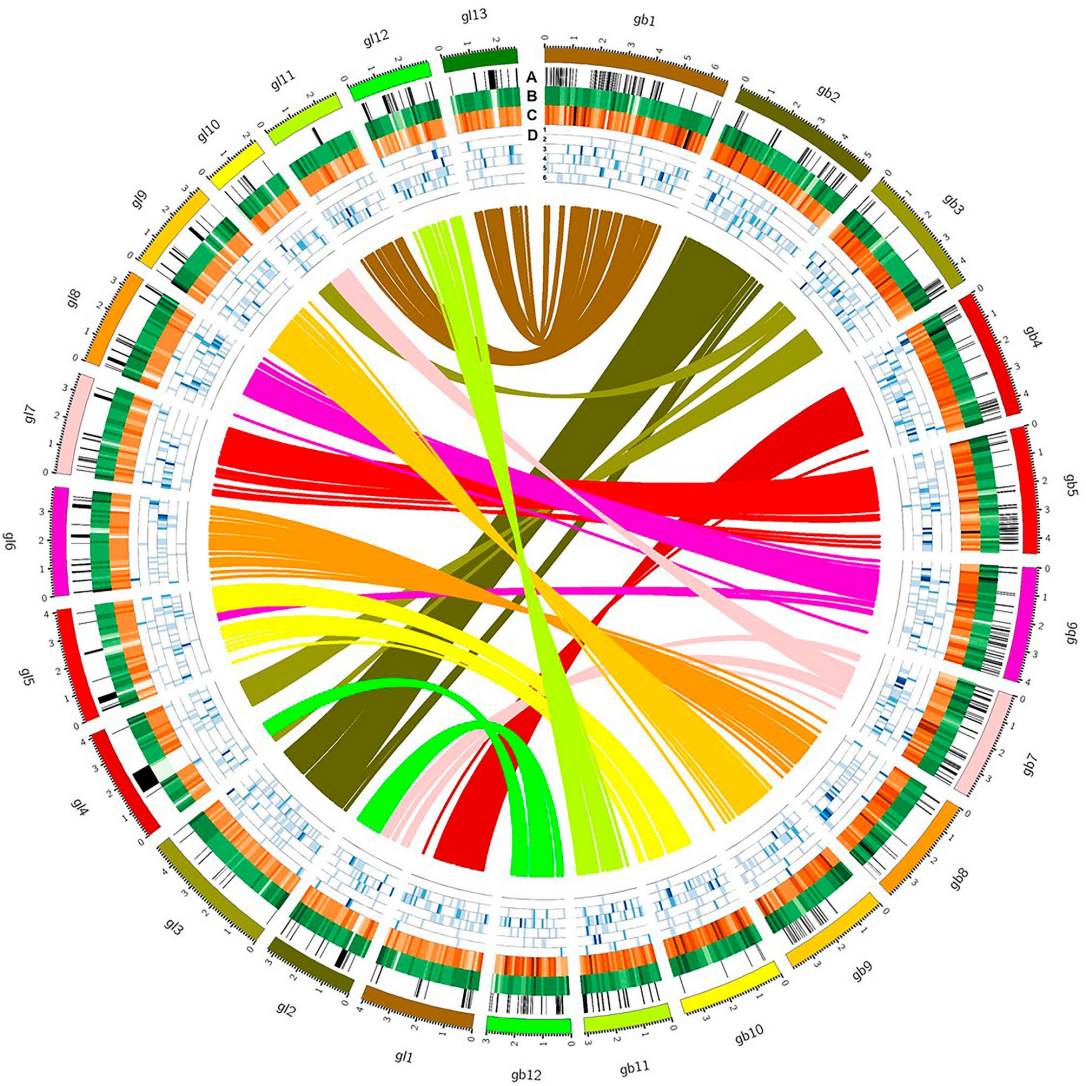
Circos plot of the genomic features of *Gb* versus *Gl*. The circles from outside to inside represent the chromosome map, sequence gaps (**A**), GC content (**B**), gene density (**C**), and CAZys content of the six families; AA (**D1**), CBM (**D2**), CE (**D3**), GH (**D4**), GT (**D5**), and PL (**D6**). Syntenic gene pairs are linked by colored lines in the inner-most circle. Each feature was calculated based on 100 kb nonoverlapping windows.

*Ganoderma* Karst. (Ganodermataceae, Polyporales) is a cosmopolitan genus of white rot fungi with more than 200 members^41,42^. The two best-known taxa economically play ecological niches either as plant pathogens for various tree crops (*Gb*) or as commercial edible herbal medicines (*Gl*)^54^. Previously, three whole-genome sequence projects of *Ganoderma* species were reported, each for *Gl*, *Ganoderma* sp., and *G. sinense*^16,55,56^. The first and last mentioned fungi are known for their medicinal properties, while *Ganoderma* sp. was described as a forest saprophyte. However, *Gl* is more popular and well characterized, providing us with the opportunity to appraise any genomic discrepancy that may underlie the different ecological niches and perhaps to identify pathogenicity factors in *Gb*. Moreover, the available high-quality genome assemblies of *Gb* and *Gl*, which are early-sequenced *Ganoderma* fungi, facilitate pairwise comparisons of genomic features and specific components (Fig. 8).

Both *Gb* and *Gl* are white-rot fungi that degrade plant cell walls via lignocellulose depolymerization^57^. A side-by-side comparison between both species revealed that the number of copies of CAZY-encoding genes in *Gb* was greater than that in *Gl*. Most likely, this study may describe a complex mechanism underpinning divergent ecological niches between *Gb* as a phytopathogen and *Gl* as a saprobe. A richer repertoire of CAZYs observed in *Gb* is likely needed to enable the degradation of living woody tissues of oil palm, whereas two-thirds of oil palm trunk biomass is composed of lignocellulose (17.1% lignin, 41.2% cellulose, and 34.4% hemicellulose)^58^. As commonly found in other fungi, both fungal species also contain the GH family, which has the largest number of CAZys, and many of the enzymes play a crucial role in the hydrolysis of carbon‒oxygen‒carbon bonds and hereinafter break sugar residues and utilize them for consumption^59^. Additionally, the complete set of ligninolytic enzymes comprises phenol oxidase CAZy AA1 (laccases: EC 1.10.3.2) and peroxidase CAZy AA2 (lignin peroxidase/EC 1.11.1.14; manganese peroxidase/EC 1.11.1.13; and versatile peroxidase/EC 1.11.1.16) to catalyze the oxidation of phenolic units in lignin, and free phenoxy radicals are present in both fungi^60^. This is because fungal laccases and peroxidases are involved in the depolymerization of lignin, defense or protection against antimicrobial effects, virulence and/or phytopathogenicity^61^.

## Methods

### Fungal strain

We used a *Gb* strain from our collection cultures labeled the G3 strain. Previously, this strain was used to generate a draft genome^32^. The fungus was maintained in presterilized rubber wood sawdust (RWS) culture in the dark at 28 °C and subcultured every 6 months. Approximately 10 mg of RWS was revived on potato dextrose agar (PDA) for 7 days each time prior to use. Freshly pure revived mycelia were grown in 100 ml of yeast malt broth in the dark at 28 °C for 14 days. Mycelia were harvested on a layer of Whatman paper no. 1 and air-dried for 15 min. Genomic DNA was processed using a GenElute Plant Genomic DNA Miniprep Kit (Sigma–Aldrich Co., St. Louis, MO, USA) according to the manufacturer’s instructions for PacBio and Illumina platform sequencing. Freeze-dried mycelia samples were shipped to Dovetail Genomic, Inc., USA, for Chicago and Hi-C library preparation.

### Illumina/PacBio sequencing and de novo assembly

Single-molecule sequencing was performed using PacBio RS II with the latest P6-C4 chemistry systems and an Illumina HiSeq 4000 system according to each manufacturer’s instructions. The PacBio reads were assembled using WTDBG2 with default parameters^34^. Subsequently, two rounds of Racon were used for consensus calling for this assembly. Running a Racon for long reads, two or several rounds, can increase the accuracy of bridging areas between contigs, generate high-quality consensus sequences, and achieve further iterations of the consensus sequence^35^. At the final assembly step, two rounds of PacBio-Racon assemblies were polished by basecall correction through Pilon software with default parameters^36^. Pilon uses Illumina reads to perform base corrections and derive an accurate consensus sequence. Moreover, at least two rounds of pilon polishing are required to achieve the same level of accuracy in most cases^62^.

### Chicago consolidant library preparation and sequencing

The Chicago library preparation and sequencing were outsourced to Dovetail Genomics (Chicago, USA) and performed as follows. Briefly, ~ 500 ng of high-molecular-weight genomic DNA (gDNA) (mean fragment length = 59) was reconstituted into chromatin in vitro and fixed with formaldehyde. The fixed chromatin was digested with *Dpn*II, the 5’ overhangs were filled with biotinylated nucleotides, and the free blunt ends were subsequently ligated. After ligation, the crosslinks were reversed, and the DNA was purified from the protein. The purified DNA was treated to remove biotin that was not internal to the ligated fragments. The DNA was then sheared to an ~ 350 bp mean fragment size, and sequencing libraries were generated using NEBNext Ultra enzymes and Illumina-compatible adapters. Biotin-containing fragments were isolated using streptavidin beads before PCR enrichment of each library. The libraries were sequenced on an Illumina HiSeq X platform to produce 165 million 2 × 150 bp paired-end reads, which provided 1887.11 × physical coverage of the genome (1–100 kb pairs).

### Hi-C library preparation and sequencing

Hi-C library preparation and sequencing were also performed by Dovetail Genomics (Chicago, USA) as follows. Briefly, for each library, chromatin was fixed in place with formaldehyde in the nucleus and then extracted. The fixed chromatin was digested with *Dpn*II, the 5ʹ overhangs were filled with biotinylated nucleotides, and the free blunt ends were subsequently ligated. After ligation, the crosslinks were reversed, and the DNA was purified from the protein. The purified DNA was treated to remove biotin that was not internal to the ligated fragments. The DNA was then sheared to an ~ 350 bp mean fragment size, and sequencing libraries were generated using NEBNext Ultra enzymes and Illumina-compatible adapters. Biotin-containing fragments were isolated using streptavidin beads before PCR enrichment of each library. The libraries were sequenced on an Illumina HiSeq X platform to produce 194 million 2 × 150 bp paired-end reads, which provided 41,650.61× physical coverage of the genome (10–10,000 kb pairs).

### Scaffolding the assembly with HiRise

The input de novo assembly, shotgun reads, Chicago library reads, and Dovetail Hi-C library reads were used as input data for HiRise, a software pipeline designed specifically for using proximity ligation data to scaffold genome assemblies^29^. An iterative analysis was conducted. First, the shotgun and Chicago library sequences were aligned to the draft input assembly using a modified SNAP read mapper (http://snap.cs.berkeley.edu). The separations of Chicago read pairs mapped within draft scaffolds were analyzed by HiRise to produce a likelihood model for genomic distance between read pairs, and the model was used to identify and break putative misjoins, to score prospective joins, and to make joins above a threshold. After aligning and scaffolding the Chicago data, Dovetail Hi‒C library sequences were aligned and scaffolded following the same method. After scaffolding, the shotgun sequences were used to close gaps between contigs.

### Gene annotation

Protein-coding gene prediction was performed using the de novo prediction tool BRAKER with the default parameters^59^. It is a novel automated protein-coding gene predictor program that works through a combination of GeneMark-ET^60^ for protein-coding gene prediction and is used as a training set for the AUGUSTUS pipeline^61^. GeneMark-ET was subjected to RNA-seq read alignment to assemble the RNA-seq transcripts of *Gb* derived from in vitro growth in rich potato dextrose broth media for 14 days. This transcript evidence was confirmed by reference to the available reference protein of *G. sinense*^52^.

All the predicted Gb transcripts were annotated against the UniProtKB/Swiss-Prot database using BLASTX software with a cutoff of an E-value restriction of 10^–6^^34^. The annotations of the sequences with the best scores were chosen to be the annotations of the genes according to the functional term classification of each database. The GO enrichment analysis was performed using the enricher function from the PANNZER, while KO, COG, and PHI analyses were each performed using TRAPID 2.0, EggNOG 5.0, and PHI-base according to their default parameters^62–65^. The annotations for ABC transporters, CAZys, and transcription factors were performed using HMMer (version 3.0) based on each particular motif in the PANTHER database^66^.

### Variants and calling SNPs and short INDELs

The Illumina reads were aligned to the reference genome of *Gb* using BWA-MEM^63^. Variants with a minimum quality > 30 were called using BCFtools (mpileup and call)^64^. Variants with a depth < 20 reads, minor allele frequency (MAF) < 0.05, missing data < 10%, and number of alleles > 2 were removed using VCFtools^65^.

### Genome comparison analysis

Syntenic genes between *Gb* and *Gl* were identified using LASTZ^66^. Comparative genome analysis was carried out by comparing genome characteristics, including CAZys, which are characteristic of white-rot fungi, with the previously reported genome sequence of *Gl*^16^. All putative *Gb* proteins were searched against entries in the CAZy database (http://www.cazy.org/) using BLASTP. The proteins with e-values smaller than E-06 were further screened by a combination of BLAST searches against individual protein domains belonging to the AA, CBM, CE, GH, GT, and PL class motifs. HMMer (version 3.0) was used to query against a collection of custom-made hidden Markov model (HMM) profiles constructed for each CAZy class. All identified proteins were then manually curated.

### Ethics declaration

All the authors declare that plant collection and use were in accordance with all the relevant guidelines.

### Plant material permission

All authors state appropriate permissions and/or licenses for collection of plant or seed specimens used in this study.

### Supplementary Information

Supplementary Tables.

## Data Availability

The whole-genome sequence data of this study has been deposited at DDBJ/EMBL/GenBank (http://www.ncbi.nlm.nih.gov/) under the accession no. PJEW03000000 version.
